# Safety Assessment of Perioperative Pain Medications for Children: Variation in Opioid Prescribing at Discharge

**DOI:** 10.1097/AS9.0000000000000664

**Published:** 2026-05-13

**Authors:** Mallory N. Perez, Lynn Huang, Willemijn L.A. Schäfer, Alison J. Lehane, Charesa J. Smith, Sarah Kennedy, Jane L. Holl, Charles J. Aprahamian, Srikumar B. Pillai, Bethany J. Slater, Mehul V. Raval

**Affiliations:** From the * Division of Pediatric Surgery, Department of Surgery, Northwestern University Feinberg School of Medicine, Ann & Robert H. Lurie Children’s Hospital of Chicago, Chicago, IL; † Center for Health Services and Outcomes Research, Institute of Public Health and Medicine, Northwestern University Feinberg School of Medicine, Chicago, IL; ‡ Department of Neurology, Biological Sciences Division and Center for Healthcare Delivery Science and Innovation, University of Chicago, Chicago, IL; § Division of Pediatric Surgery, Department of Surgery, OSF Healthcare, Peoria, IL; ¶ Division of Pediatric Surgery, Department of Surgery, RUSH Medical College, Chicago, IL; ‖ Division of Pediatric Surgery, Department of Surgery, University of Chicago, Chicago, IL.

**Keywords:** opioid, pain management, quality improvement, pediatric, surgery

## Abstract

**Objective:**

To characterize factors contributing to opioid prescribing practice variation at discharge for children after surgery and evaluate the association between discharge opioid prescribing and 30-day follow-up/complications.

**Background:**

Efforts to optimize opioid use in children after surgery are currently limited by insufficient information about prescribing practice variation. This paper comprehensively examines discharge opioid prescribing practices across pediatric surgical specialties and hospitals.

**Methods:**

Prospective cohort study of 1670 children (5–17 years) from 4 Illinois hospitals participating in the National Surgical Quality Improvement Program-Pediatric, using electronic health record data abstracted from January 2021 to April 2023. Primary outcome measures were opioid exposure (receiving an opioid prescription at discharge) and opioid dose intensity (total morphine milligram equivalents [MMEs] prescribed) at discharge. Associations between each of the primary outcomes and patient/clinical factors were evaluated with multivariable logistic and multivariable linear regressions.

**Results:**

In total, 566 (34%) children had an opioid exposure with median dose intensity 80 MMEs/prescription (interquartile range: 50–125) at discharge. Hospital site, older age (Odds ratio [OR], 2.78 [95% confidence interval (CI), 2.03–3.82]; β = 47.12, *P* < 0.001), preoperative non-opioid analgesia use (OR, 2.42 [95% CI, 1.63–3.62]; β = 26.84, *P* < 0.001), and regional anesthesia use (OR, 10.14 [95% CI, 5.12–20.02]; β = 25.77, *P* = 0.03 ) were associated with opioid exposure and dose intensity. Surgical specialties with increased opioid exposure did not correspond with those with higher dose intensity. Lack of opioid exposure at discharge was not associated with pain control issues requiring follow-up care.

**Conclusions:**

Significant variation by hospital and surgical specialty exists in opioid exposure and dose intensity for children after surgery. Both opioid exposure and dose intensity offer valuable, complementary insights, and therefore, should be monitored to fully optimize opioid stewardship in children after surgery.

## INTRODUCTION

The opioid epidemic of the 2000s has had a substantial impact on all populations, including children and adolescents.^1,2^ Despite great strides in addressing the epidemic, opioid prescribing rates are still higher in the United States (US) than in other high-income countries.^3,4^ Childhood exposure to opioids for postoperative analgesia is a common route to opioid misuse,^5^ and prescription opioid use during adolescence is a risk factor for persistent use and future misuse.^6,7^ Since over 3 million children and adolescents undergo surgery in the US annually, opioid prescribing for these children at hospital discharge can be highly impactful, especially for those who are opioid-naïve.^8,9^ Moreover, evidence suggests that surgeons frequently prescribe more opioids at hospital discharge than needed, leading to a surplus of unused opioids potentially accessible for nonmedical use.^10–15^ Certainly, opioid stewardship efforts must balance adequate pain control with risks of opioid-related harm.

Opioid prescribing in children remains inadequately studied and, potentially, underestimated.

For certain procedures (eg, umbilical hernia repair) and pediatric surgical specialties (eg, urology), dosing recommendations and surgeon education have successfully reduced postoperative opioid prescribing.^16,17^ However, such recommendations are underused and have not been comprehensively implemented in pediatric surgery.^15,18–20^ The limited uptake of interventions to minimize opioid use has been perpetuated by an incomplete understanding of hospital- and pediatric surgical specialty-level variation in discharge opioid prescribing.

In 2022, Ingram et al published a novel, prospective, cohort study that leveraged data from the American College of Surgeons National Surgical Quality Improvement Program-Pediatric (NSQIP-Pediatric), a national registry for quality improvement initiatives, to evaluate pediatric perioperative pain management practices at a single, free-standing children’s hospital.^21^ The study demonstrated the feasibility of combining patient-level NSQIP-Pediatric data and 19 additional electronic health record (EHR)-abstracted variables about pain and analgesia. The study also identified factors associated with higher opioid prescribing at discharge (eg, adolescent age, White race, and orthopedic surgery).

Building upon this work, the Safety Assessment of Perioperative Pain medications for children study conducted a similar cohort study, leveraging NSQIP-Pediatric and additional EHR-abstracted variables about pain and analgesia to investigate pain management practices at discharge across 4 hospitals in Illinois. We hypothesized that opioid prescribing at discharge varies significantly by hospital and surgical specialty, both in the proportion of patients receiving opioid prescriptions (exposure) and the total amount of opioid prescribed per patient (dose intensity) at discharge.

## METHODS

### Setting and Patient Sample

The Safety Assessment of Perioperative Pain medications for children study is a multicenter, prospective, cohort study of patients who underwent surgery at 4 children’s hospitals from January 2022 to April 2023. The sites represent all children’s hospitals participating in NSQIP-Pediatric in Illinois. Patients selected for NSQIP-Pediatric data abstraction, ages 5 to 17 years, were eligible for inclusion. Patients who underwent concurrent procedures for different indications or who had a length of stay over 30 days were excluded, per NSQIP-Pediatric criteria. Patients who were non-English- or non-Spanish-speaking were also excluded.

The NSQIP-Pediatric sampling framework encompasses a diverse range of pediatric surgical procedures, both inpatient and outpatient, across various pediatric surgical specialties. To mitigate bias toward any specific procedure within a hospital cohort, strategic case selection occurs on an 8-day rotation. Certified Surgical Clinical Reviewers (SCRs) abstract patient-level clinical variables from EHRs into the NSQIP-Pediatric database. Institutions must have adequate annual surgical case volumes to participate in NSQIP-Pediatric. For this study, SCRs prospectively screened cases, identifying any case occurring during the 8-day rotation anticipated to be eligible for NSQIP-Pediatric based on the program’s inclusion criteria.

### Data Extraction and Abstraction

As described by Ingram et al,^21^ additional EHR-abstracted variables related to pain management were collected and combined with selected American College of Surgeons NSQIP-Pediatric variables (Supplemental Table 1, see https://links.lww.com/AOSO/A594). A Research Electronic Data Capture (REDCap) database, hosted at Northwestern University, was created for data entry using standard NSQIP-Pediatric abstraction practices.^22,23^ Each case selected by the SCRs through prospective screening was assigned a unique de-identified REDCap ID number. Biweekly meetings were held between the research team and the hospitals’ SCRs to address any questions regarding the REDCap database.

### Outcome(s) of Interest

The primary outcomes of interest were (1) opioid exposure at hospital discharge (hereafter “discharge opioid exposure”), defined as the prescription of an opioid (Y/N) by any clinician at discharge from the index surgery, and (2) dose intensity, defined as total morphine milligram equivalents (MMEs) of opioid prescribed at discharge. MME per day is a standardized way to describe the quantity of opioid prescribed over a 24-hour period, often used to evaluate risk of polypharmacy, misuse, and overdose.^24^ The MME/day was calculated using opioid type (eg, oxycodone and hydrocodone), form (eg, pill and liquid), unit dose, and dose frequency, using the standard centers for disease control and prevention morphine milligram equivalent conversion factors. MME/day was then multiplied by the prescription duration (days) to calculate total MME.^25^ The calculation of dose intensity does not reflect the actual total MME taken by the patient, but rather the total MME prescribed and potentially available to the patient.

As a balancing measure, EHR-abstracted post-discharge follow-up encounters for postoperative pain within 30 days of surgery (eg, telephone/email, clinic, and urgent/emergent care) and refills or new opioid prescriptions prescribed up to 30 days postoperatively were recorded in REDCap. NSQIP-Pediatric data on 30-day unplanned hospital readmission and postoperative complications, measured as a composite of 14 variables, were also evaluated.

### Covariates

Patient-level demographics and clinical data, including perioperative analgesic use, as well as surgery-specific data were abstracted. Demographic variables included: age, sex, race (White, Black/African American, Asian/Pacific Islander, and Other), and ethnicity. Clinical variables included: American Society of Anesthesiologists (ASA) classification, history of substance (tobacco, alcohol, marijuana, opioid, or other illicit substance) misuse, and an active opioid prescription from a prior encounter (other than the surgical encounter) at the time of abstraction (eg, a prescription from a prior ED visit). Additional data on pharmacologic pain management strategies included: use of preoperative non-opioid analgesia (eg, oral/intravenous acetaminophen, gabapentinoid, and lidocaine patch), use of regional/local anesthesia (eg, epidural/caudal, spinal, peripheral nerve, transversus abdominis plane (TAP) blocks, cryoablation, and peri-incisional infiltration), prescription of non-opioid analgesia at discharge, and having an active prescription for a benzodiazepine at discharge. Preoperative medication administration was defined as any medication administered on the day of surgery prior to the initiation of anesthesia, regardless of care setting. Surgery-specific data included surgical length of stay, operative approach (open versus laparoscopic), and surgical specialty.

### Statistical Analysis

Demographic characteristics were reported as mean (SD), median (interquartile range [IQR]), or percentage (number), as appropriate. Chi-square tests for categorical variables (eg, sex, specialty, and yes/no discharge opioid prescription) and Kruskal–Wallis tests for continuous variables (eg, total MME) were used for comparisons across the 4 hospitals. For 30-day postoperative complications, univariate analysis was performed using chi-square or Fisher exact tests to evaluate the association between discharge opioid exposure (Y/N) and complications. Statistical significance was set at *P* < 0.05.

To further explore hospital-level variation in discharge opioid exposure, we performed a multivariable logistic regression with a fixed effect for hospital site. To examine variation in opioid dose intensity, a multivariable linear regression was performed, also with a fixed effect for hospital site.

After observing the variation in discharge opioid exposure by surgical specialty, we conducted bivariate sub-analyses using chi-square tests by hospital and age group (children: 5–11 years old, adolescents: 12–17 years old). The significance threshold, adjusted using a Bonferroni correction, was established at *P* < 0.006. The relative magnitude of difference (RMD) was calculated to compare the magnitude of variation by hospital and by age group. We defined RMD as the ratio of the absolute difference in discharge opioid exposure for the lowest and highest prescribing groups to discharge opioid exposure for the lowest prescribing group, multiplied by 100. For example, if discharge opioid exposure ranged from 5% to 20% across hospitals, the absolute difference was 0.2 − 0.05 = 0.15, which when divided by the lowest opioid exposure rate (0.05), equaled 3, and was multiplied by 100 for a RMD of 300%. This ratio represents the variation of discharge opioid exposure across the 4 hospitals for a given surgical specialty. A similar analysis was performed by age group. In addition to the *P* value, this common variable allows for relative comparisons across categories.

All statistical analyses were conducted using SAS 9.4 (Cary, NC). The study was approved by the Institutional Review Board of Ann & Robert H. Lurie Children’s Hospital of Chicago with expedited review for all participating sites (IRB 2021-4519).

## RESULTS

### Characteristics of the Study Cohort

**FIGURE 1.**
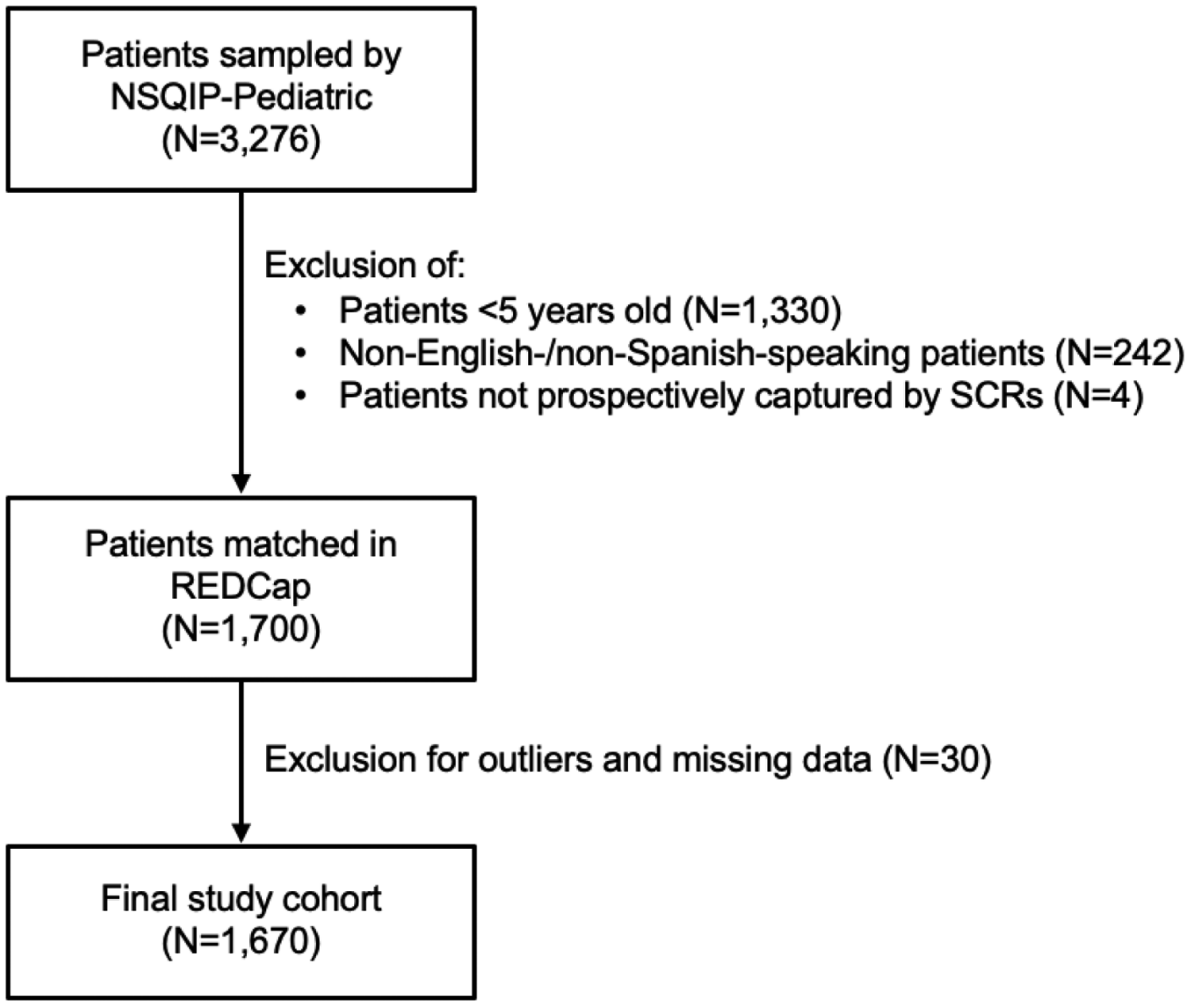
Flow diagram of study population. The cohort selection for the Safety Assessment of Perioperative Pain medications for children (SAPPhire) study. Of 3276 patients sampled by NSQIP-Pediatric, exclusions included age <5 years (N = 1330), non-English/non-Spanish speakers (N = 242), and patients not prospectively captured by surgical clinical reviewers (N = 4). After matching in REDCap (N = 1700), 30 patients were excluded for missing data, resulting in a final analytic cohort of 1670 children.

In total, 3276 patients were identified in NSQIP-Pediatric at the 4 Illinois hospitals. Of those patients, 1700 (45.6%) met the study’s inclusion criteria and were captured in the REDCap database. Thirty patients (1.8%) were excluded due to missing data. The final cohort consisted of 1670 patients (Fig. 1).

Descriptive statistics of the study population characteristics are provided in Table 1. A summary of outcome measures is presented in Table 2. Less than 2% (N = 31) of patients had an active opioid prescription from a prior encounter, and 1% (N = 19) had a history of substance misuse. Overall, 34% (N = 566) of patients had a discharge opioid exposure. Median dose intensity was 80 MMEs (IQR: 50–125). Most prescriptions were for oxycodone (N = 350, 62%) and written for greater than or equal to a 3-day supply (N = 313, 55%). Few patients were prescribed a mean daily MME > 50 (N = 33, 6%). Less than 1% (N = 15) of patients received a new prescription or refill after discharge.

**TABLE 1.** Descriptive Statistics by Hospital (N = 1670)

	Hospital 1	Hospital 2	Hospital 3	Hospital 4	Overall	*P*
	289 (17.3%)	114 (6.8%)	284 (17.0%)	983 (58.9%)	1670 (100%)	
Gender						0.33
Male	152 (52.6)	54 (47.4)	134 (47.2)	529 (53.8)	869 (52.0)	
Female	136 (47.1)	60 (52.6)	150 (52.8)	450 (45.8)	796 (47.7)	
Nonbinary	1 (0.4)	0	0	4(0.40)	5 (0.3)	
Age						**0.02**
Children (5–11)	143 (49.5)	41 (36.0)	130 (45.8)	412 (41.9)	729 (43.6)	
Adolescents (12–17)	146 (50.5)	73 (64.0)	154 (54.4)	571 (58.1)	941 (56.4)	
Race						**<0.001**
White	232 (80.3)	49 (43.0)	88 (31.0)	467 (47.5)	836 (50.1)	
Black	31 (10.7)	20 (17.5)	114 (40.1)	89 (9.0)	254 (15.2)	
Asian	2 (0.7)	2 (1.8)	9 (3.2)	38 (3.9)	51 (3.0)	
Other/unknown	24 (8.3)	43 (37.7)	73 (25.7)	389 (39.6)	529 (31.7)	
Ethnicity						**<0.001**
Hispanic	20 (6.9)	54 (47.4)	67 (23.6)	368 (37.4)	509 (30.5)	
Not Hispanic	267 (92.4)	58 (50.9)	193 (68.0)	585 (59.5)	1103 (66.0)	
Unknown	2 (0.7)	2 (1.7)	24 (8.4)	30 (3.1)	58 (3.5)	
Insurance						**<0.001**
Public	146 (50.5)	72 (63.2)	154 (54.2)	54 (5.5)	426 (25.5)	
Private	137 (47.4)	42 (36.8)	129 (45.4)	925 (94.1)	1233 (73.8)	
Other	6 (2.1)	0	1 (0.4)	4 (0.4)	11 (0.7)	
Specialty						**<0.001**
General surgery	150 (51.9)	47 (41.2)	134 (47.2)	415 (42.2)	746 (44.7)	
Gynecology	10 (3.5)	3 (2.6)	14 (4.9)	—	27 (1.6)	
Neurosurgery	20 (6.9)	4 (3.5)	24 (8.5)	114 (11.6)	162 (9.7)	
Otolaryngology (ENT)	21 (7.3)	28 (24.6)	30 (10.6)	132 (13.4)	211 (12.6)	
Orthopedic	74 (25.6)	18 (15.8)	43 (15.1)	177 (18.0)	312 (18.7)	
Plastics	2 (0.7)	12 (10.5)	19 (6.7)	51 (5.2)	84 (5.0)	
Urology	12 (4.1)	2 (1.8)	20 (7.0)	94 (9.6)	128 (7.7)	
Surgical approach						**<0.001**
Laparoscopic	158 (54.7)	45 (39.5)	147 (51.8)	439 (44.7)	789 (47.3)	
Open/unclassified	131 (45.3)	69 (60.5)	137 (48.2)	544 (55.3)	881 (52.7)	
ASA classification						**<0.001**
ASA 1	74 (25.6)	44 (38.6)	107 (37.7)	246 (25.0)	471 (28.2)	
ASA 2	145 (50.2)	52 (45.6)	118 (41.5)	473 (48.1)	788 (47.2)	
ASA 3	56 (19.4)	16 (14.0)	48 (16.9)	258 (26.3)	378 (22.6)	
ASA 4	14 (4.8)	2 (1.8)	11 (3.9)	6 (0.6)	33 (2.0)	
Preoperative non-opioid analgesia						**<0.001**
Yes	174 (60.2)	12 (10.5)	116 (40.9)	152 (15.5)	454 (27.2)	
No	115 (39.8)	102 (89.5)	168 (59.1)	831 (84.5)	1216 (72.8)	
Anesthetic block						**<0.001**
Regional only	0	17 (14.9)	3 (1.1)	179 (18.2)	199 (11.9)	
Local only	247 (84.5)	60 (52.6)	210 (73.9)	609 (62.0)	1126 (67.4)	
Both	1(0.3)	32 (28.1)	4 (1.4)	112 (11.4)	149 (8.9)	
None	41 (14.2)	5 (4.4)	67 (23.6)	83 (8.4)	196 (11.7)	

Bold indicates *P* < 0.05.

**TABLE 2.** Summary of Outcomes by Hospital (N = 1670)

	Hospital 1	Hospital 2	Hospital 3	Hospital 4	Overall		*P*
	289 (17.3%)	114 (6.8%)	284 (17.0%)	983 (58.9%)	1670 (100%)		
LOS (med, IQR)	1 (0–2)	1 (0–2)	1 (0–3)	1 (0–3)	1 (0–3)		**<0.001**
Active benzodiazepine at discharge							**<0.001**		
Yes	5 (1.7)	18 (15.8)	16 (5.6)	242 (24.6)	281 (16.8)		
No	283 (97.9)	96 (84.2)	268 (94.4)	741 (75.4)	1388 (83.1)		
Missing	1 (0.4)	0	0	0	1 (0.1)		
Non-opioid analgesic at discharge							**<0.001**		
Yes	236 (81.7)	91 (79.8)	200 (70.4)	800 (81.4)	1327 (79.5)		
No	53 (18.3)	23 (20.2)	84 (29.6)	182 (18.5)	342 (20.5)		
Missing	0	0	0	1 (0.1)	1 (0.1)		
Opioid prescribed at discharge							**<0.001**		
Yes	72 (24.9)	51 (44.7)	82 (28.9)	361 (36.7)	566 (33.9)		
No	217 (75.1)	63 (55.3)	202 (71.1)	622 (63.3)	1104 (66.1)		
Total days* (x, SD)	3.5 (3.0)	3.9 (3.2)	3.1 (2.7)	3.1 (1.5)	3.2 (2.2)		0.06
Total MME* (med, IQR)	75 (37.5–106)	75 (50–105)	50 (35.6–100)	90 (59–150)	80 (50–125)		**<0.001**
Daily MME* (x, SD)	26.8 (15.7)	26.1 (11.6)	26.1 (12.0)	33.8 (13.8)	31.0 (14.1)		**<0.001**
Daily MME ≥50*							0.08		
<50	71 (98.6)	49 (96.1)	80 (97.6)	333 (92.2)	533 (94.2)		
≥50	1 (1.4)	2 (3.9)	2 (2.44)	28 (7.8)	33 (5.8)		
Script ≥3 days*							**<0.001**
<3 days	36 (50.0)	22 (43.1)	50 (61.0)	145 (40.2)	253 (44.7)		
≥3 days	36 (50.0)	29 (56.9)	32 (39.0)	216 (59.8)	313 (55.3)		
Script >7 days*							**<0.001**
<7 days	68 (94.4)	44 (86.3)	75 (91.5)	358 (99.2)	545 (96.3)		
≥7 days	4 (5.6)	7 (13.7)	7 (8.5)	3 (0.8)	21 (3.7)		

Bold indicates *P* < 0.05.

* Calculated for patients with a discharge opioid exposure (N = 566).

LOS, length of stay.

With regard to the balancing measures, 127 (7.6%) patients sought follow-up care for pain, and 77 (4.6%) patients experienced at least 1 unplanned readmission within 30 days after surgery. Discharge opioid exposure was not associated with seeking follow-up care (eg, ED/urgent care visit, phone call/email, and outpatient office visit) for pain-related issues (odds ratio [OR], 1.43 [95% confidence interval (CI), 0.99–2.07]). However, discharge opioid exposure was associated with significantly lower odds of unplanned readmission for any indication, not limited to those for inadequately controlled pain (OR, 0.45 [95% CI, 0.26–0.76]).

Less than 5% (N = 79) of patients experienced a postoperative complication (Table 3). Patients with discharge opioid exposure had lower odds of experiencing any postoperative complication (OR, 0.40 [95% CI, 0.22–0.72]). Thirty-six (2.1%) patients required a subsequent surgery; however, discharge opioid exposure was not associated with reoperation (OR, 0.75 [95% CI, 0.36–1.57]). The study was underpowered to assess association between discharge opioid exposure and 30-day mortality (N = 2, 0.1%).

**TABLE 3.** Association Between NSQIP-Pediatric Complications and Opioids at Discharge

Complication*	No. (%)Total Cohort (N = 1670)	No. (%) With Discharge Opioid (N = 566)	*P* ^ †^
Superficial incisional SSI	17 (1.0%)	5 (0.9%)	0.69
Deep incisional SSI	1 (0.1%)	1 (0.2%)	0.16
Organ/deep space SSI	33 (2.0%)	5 (0.9%)	**0.02** ‡
Wound dehiscence	1 (0.1%)	1 (0.2%)	0.34
Pneumonia	3 (0.2%)	1 (0.2%)	1
Unplanned intubation	2 (0.1%)	1 (0.2%)	0.63
Mechanical ventilation >48 h	18 (1.1%)	2 (0.4%)	**0.04** ‡
Renal insufficiency	2 (0.1%)	1 (0.2%)	1
Urinary tract infection	2 (0.1%)	0	0.55
Cardiac arrest	1 (0.1%)	0	1
Deep vein thrombosis	1 (0.1%)	0	1
Sepsis	3 (0.2%)	2 (0.4%)	0.27
Septic shock	3 (0.2%)	0	0.56
Central line infection	1 (0.1%)	0	1
Any complication (yes/no)	79 (4.7%)	14 (2.5%)	**0.002** ‡

Bold indicates *P* < 0.05.

* No patients experienced postoperative coma, clostridium difficile infection, nerve injury, seizure, intraventricular hemorrhage, or required postoperative dialysis.

† *P* value represents significance of chi-square or Fishers test, as appropriate by count volume.

‡ Indicates statistically significant negative association between complication and receipt of discharge opioid.

### Variation in Discharge Opioid Exposure

To identify factors associated with discharge opioid exposure, we performed a multivariable logistic regression (Table 4). Hospitals differed significantly in discharge opioid exposure; for example, Hospital 1 had 75% lower odds of discharge opioid exposure (OR, 0.25 [95% CI, 0.14–0.44]) compared to Hospital 4. Adolescents, compared to younger children, had higher odds of discharge opioid exposure (OR, 2.78 [95% CI, 2.03–3.82]). Children undergoing gynecologic (OR, 35.65 [95% CI, 12.86–98.8]), orthopedic (OR, 50.30 [95% CI, 28.28–89.46]), plastic (OR, 12.46 [95% CI, 6.40–24.23]), or urologic (OR, 3.32 [95% CI, 1.96–5.62]) surgery, independently, had increased odds of discharge opioid exposure compared to those undergoing general surgery. Patients who received a preoperative non-opioid analgesic (OR, 2.42 [95% CI, 1.63–3.62]) or who received local/regional anesthesia (OR, 10.14 [95% CI, 5.12–20.02]) had higher odds of discharge opioid exposure compared to those who did not. The following covariates were not associated with discharge opioid exposure: gender, race, insurance, ASA classification, neurosurgery, and otolaryngology (*P* > 0.05).

**TABLE 4.** Multivariable Logistic Regression for Opioid Prescribed at Discharge

Variable	OR	95% CI	*P*
Site			
Hospital 1	0.25	(0.14, 0.44)	**<0.001**
Hospital 2	1.27	(0.70, 2.31)	0.42
Hospital 3	0.65	(0.40, 1.05)	0.08
Hospital 4	Reference		
Gender			
Male	Reference		
Female	0.95	(0.71, 1.28)	0.75
Nonbinary	0.65	(0.04, 9.81)	0.75
Age			
Children (5–11)	Reference		
Adolescents (12–17)	2.78	(2.03, 3.82)	**<0.001**
Race			
White	Reference		
Black	1.04	(0.66, 1.65)	0.86
Asian	0.76	(0.33, 1.76)	0.53
Other	1.03	(0.66, 1.60)	0.91
Ethnicity			
Hispanic	0.56	(0.36, 0.88)	**0.01**
Non-Hispanic	Reference		
Unknown	0.80	(0.34, 1.89)	0.61
Insurance			
Public	Reference		
Private	1.37	(0.90, 2.09)	0.15
Other	0.81	(0.11, 6.03)	0.84
Specialty			
General surgery	Reference		
Gynecology	35.65	(12.86, 98.8)	**<0.001**
Neurosurgery	1.04	(0.57, 1.89)	0.89
Orthopedics	50.30	(28.28, 89.46)	**<0.001**
Otolaryngology (ENT)	1.50	(0.90, 2.51)	0.12
Plastics	12.46	(6.40, 24.23)	**<0.001**
Urology	3.32	(1.96, 5.62)	**<0.001**
Surgical approach			
Laparoscopic	Reference		
Open/unclassified	2.51	(1.68, 3.77)	**<0.001**
ASA classification			
ASA 1	Reference		
ASA 2	1.35	(0.95, 1.92)	0.10
ASA 3	1.03	(0.63, 1.62)	0.91
ASA 4	0.82	(0.24, 2.78)	0.75
Preoperative non-opioid analgesia			
Yes	2.42	(1.63, 3.62)	**<0.001**
No	Reference		
Anesthetic block			
Regional only	2.83	(1.48, 5.43)	**0.002**
Local only	2.04	(1.24, 3.36)	**0.01**
Both	10.14	(5.13, 20.02)	**<0.001**
None	Reference		

Bold indicates *P* < 0.05.

### Variation in Opioid Dose Intensity at Discharge

To assess variation in dose intensity, we performed a multivariable linear regression, limited to the 566 patients with a discharge opioid exposure. The model demonstrated moderate explanatory power (*R*^2^ = 0.31) (Table 5). Adolescents (*P* < 0.001) and females (*P* = 0.03) had significantly higher dose intensities. Patients who underwent neurosurgery (*P* = 0.01) and orthopedic surgery (*P* < 0.001) had higher dose intensities compared to patients who underwent general surgery. Patients who underwent gynecologic surgery had a lower dose intensity, although not statistically significant (*P* = 0.51). Both preoperative non-opioid analgesia (*P* < 0.01) and local/regional anesthesia (*P* = 0.05) were significantly associated with a higher dose intensity.

**TABLE 5.** Multivariable Linear Regression for Total MME Prescribed at Discharge

Variable	Coefficient	SE	95% CI	*P*
Site				
Hospital 1	−6.26	10.07	(−26.04, 13.53)	0.53
Hospital 2	12.48	11.41	(−9.94, 34.91)	0.27
Hospital 3	−19.02	11.33	(−41.28, 3.23)	0.09
Hospital 4	Reference			
Gender				
Male	Reference			
Female	12.87	5.91	(1.26, 24.49)	**0.03**
Nonbinary	7.88	39.32	(−69.36, 85.12)	0.84
Age				
Children (5–11)	Reference			
Adolescents (12–17)	47.12	6.71	(33.93, 60.31)	**<0.001**
Race				
White	Reference			
Black	1.31	8.88	(−16.13, 18.74)	0.88
Asian	−9.66	15.84	(−40.78, 21.45)	0.54
Other	−1.29	9.00	(−18.97, 16.39)	0.87
Ethnicity				
Hispanic	−8.42	8.82	(−25.74, 8.91)	0.39
Non-Hispanic	Reference			
Unknown	14.21	16.53	(−18.26, 46.67)	0.34
Insurance				
Public	Reference			
Private	5.44	9.37	(−12.96, 23.85)	0.13
Other	−59.24	39.00	(−135.86, 17.38)	0.56
Specialty				
General surgery	Reference			
Gynecology	−11.25	18.00	(−46.62, 24.11)	0.53
Neurosurgery	44.66	15.85	(13.52, 75.80)	**0.01**
Orthopedics	60.92	10.63	(40.04, 81.80)	**<0.001**
Otolaryngology (ENT)	15.28	14.42	(−13.05, 43.62)	0.29
Plastics	9.31	13.73	(−7.66, 46.29)	0.16
Urology	5.33	14.18	(−22.51, 33.19)	0.71
Surgical approach				
Laparoscopic	Reference			
Open/unclassified	8.34	10.0	(−11.29, 27.98)	0.41
ASA classification				
ASA 1	Reference			
ASA 2	−7.38	6.95	(−21.04, 6.27)	0.29
ASA 3	−1.67	9.21	(−19.76, 16.41)	0.85
ASA 4	14.55	31.29	(−46.92, 76.02)	0.64
Preoperative non-opioid analgesia				
Yes	26.84	7.05	(12.99, 40.70)	**<0.001**
No	Reference			
Anesthetic block				
Regional only	25.77	11.60	(2.98, 48.56)	**0.03**
Local only	6.19	10.04	(−13.52, 25.91)	0.54
Both	25.09	12.59	(0.37, 49.82)	**0.05**
None	Reference			

Bold indicates *P* < 0.05.

### Comparing Opioid Outcomes by Specialty

For general surgery, 15% (N = 111) of patients had a discharge opioid exposure, the lowest rate of any specialty, yet median dose intensity (median [IQR], 90 [45–90] MMEs) was the third highest among all specialties. For orthopedic surgery, patients had high discharge opioid exposure (N = 267, 85%), with the highest median dose intensity (median [IQR], 107 [60–188] MMEs) of all specialties. For urologic surgery, patients had low discharge opioid exposure (N = 36, 28%) with the lowest median dose intensity (median [IQR], 37 [25–63] MMEs) of all specialties.

### Sub-Analyses of Specialty-level Variation by Hospital and Age Group

The 2 sub-analyses further describing surgical specialty-level variation in discharge opioid exposure, stratified by hospital and age group, are provided in Supplemental Tables 2 and 3, see https://links.lww.com/AOSO/A594. Significant specialty-level variation in discharge opioid exposure by hospital was observed for general surgery (300%, *P* < 0.001), orthopedic surgery (54%, *P* < 0.001), otolaryngology (257%, *P* = 0.001), and urology (260%, *P* = 0.004). By age group, significant variation was observed for general surgery (340%, *P* < 0.001), orthopedic surgery (29%, *P* < 0.001), and otolaryngology (400%, *P* < 0.001).

## DISCUSSION

### Clinical Significance

This study found that, on average, over one-third (33.9%) of pediatric surgical patients, captured in NSQIP-Pediatric, at 4 Illinois hospitals had a discharge opioid exposure, although with wide variation (24.9%–44.7%). Comparing this prevalence to prior pediatric surgical literature is challenging due to the heterogeneity of study populations. However, collectively, the prior studies also report wide variation in discharge opioid exposure.^21,26–28^

This study, through concurrent assessment of both discharge opioid exposure and dose intensity, offers new insights into hospital- and specialty-level variation. Notably, pediatric surgical specialties with high discharge opioid exposure may differ from those with high opioid dose intensity, underscoring the importance of considering both metrics when assessing discharge opioid prescribing. In addition, linkage of the NSQIP-Pediatric registry to additional EHR-extracted variables provided unique information about perioperative pain management as well as “balancing” measures (eg, post-discharge healthcare utilization for post-discharge pain, opioid refills) to assess potential unintended consequences of reduced discharge opioid prescribing.

Preoperative non-opioid analgesia and regional anesthesia were used modestly (27.2% and 20.8%, respectively). Perhaps paradoxically, patients who received either modality had higher odds of discharge opioid exposure. This finding is likely influenced by confounding of indication, as surgeons are more likely to use multimodal strategies, including preoperative pharmacologic agents, for procedures anticipated to be more painful. Accordingly, this finding should be interpreted as descriptive rather than causal. A recent pediatric orthopedic study similarly found that using peripheral nerve blocks during anterior cruciate ligament reconstruction in adolescents was not associated with a decrease in postoperative opioid prescriptions.^29^ Further investigation of the relationship between preoperative pharmacologic adjuncts and in-hospital opioid exposure for procedures where these non-opioid modalities are often utilized, would be beneficial. In addition, clinical nuances such as the precise timing of administration of preoperative non-opioid medications and the success rates of regional anesthetic delivery, not captured in this study, should be considered.

We acknowledge the importance of balancing the risks of opioid exposure and adequate pain control. Only 1% of patients received a new or refill opioid prescription after discharge. However, nearly 8% of patients had a post-discharge healthcare encounter (within 30 days of surgery) related to pain control. Importantly, the absence of an opioid at discharge was not associated with higher odds of seeking follow-up care for pain-related concerns. One potential explanation is that non-opioid analgesics have been found to provide equivalent pain control for many pediatric surgical procedures,^30^ and opioid prescriptions do not reduce pain intensity or the need for additional care.^31,32^ Alternative methods for post-discharge pain control (eg, non-opioid analgesics, non-pharmacologic adjuncts) employed at the time of these follow-up encounters were not assessed but remain an important area for future research.

The interpretation of postoperative complications, using the composite NSQIP-Pediatric complications variable, is challenged by the overall low rate of complications in the pediatric surgical population and the uncertain temporal relationship between hospital discharge and the occurrence of the complication. Although opioid use has known risks of respiratory (eg, aspiration) and gastrointestinal complications (eg, constipation), discharge opioid exposure was associated with lower odds of any 30-day postoperative complication. Rather than reflecting a protective effect, this finding likely points to confounding by indication and case selection. For example, analyses of NSQIP-Pediatric appendectomy data showed that patients discharged with opioids tended to have uncomplicated appendicitis, shorter lengths of stay, and fewer baseline risk factors for complications.^33^ Conversely, patients with complicated appendicitis, prolonged hospitalizations, or higher comorbidity burden may have received different discharge analgesic regimens or no prescriptions if pain was already controlled during the inpatient period. Further, patients without complications and shorter lengths of stay, may receive opioids as a “safety-net” to ensure adequate pain control after discharge. Our finding therefore warrants validation in a larger cohort, with procedure-level analysis and evaluation of specific complications rather than a composite measure.

Existing postoperative opioid prescribing guidelines are primarily based on expert consensus.^18,27,34^ To create evidence-based guidelines, specific to the pediatric surgical population, that effectively balance pain management with opioid-related harm reduction, investigation of discharge opioid exposure at the procedure level is needed. This study is the first that we are aware of that provides a method for gathering detailed pediatric perioperative pain management data by hospital and specialty and could, in the future, be applied to specific procedures. Future research should prioritize pediatric surgical specialties and procedures with the greatest discharge opioid exposure and dose intensity variation, offering great potential for improving pediatric surgery opioid stewardship on a large scale. In addition to procedure-level analyses, such studies should assess perioperative and early post-discharge pain management, including scheduled non-opioid medications, to better contextualize discharge opioid prescribing. Procedure-level analyses could further clarify findings, such as the observation that general surgery represented a low prescribing rate but relatively high dose intensity. This pattern likely reflects the heterogeneity of general surgery procedures, which spans operations with markedly different anticipated postoperative pain, ranging from, for example, umbilical hernia repair to pectus excavatum repair. Following this study, NSQIP-Pediatric incorporated many of these variables into its standard data collection for all 150 participating hospitals, advancing benchmarking endeavors.

### Limitations

There are several limitations to this study. While different hospital types (eg, urban and rural) were represented, NSQIP-Pediatric does not capture all pediatric procedures. Therefore, the discharge opioid exposure may not be fully generalizable to all pediatric procedures, specifically to those of lower acuity or those performed in the ambulatory setting. Moreover, the findings represent patients who underwent a pediatric surgical procedure at 4 hospitals with pediatric surgery programs in Illinois; national discharge opioid prescribing practices across the US remain to be fully described. Furthermore, most patients were insured and, since both geographic location and insurance status have been established as significant factors contributing to discharge opioid exposure in the combined pediatric-adult literature, these are also factors limiting the generalizability of the findings.^35^

This study reported discharge opioid exposure and did not ascertain whether the prescription was filled, and if filled, whether the child actually consumed any or all of the opioid medication. In addition, patient-reported pain outcomes (eg, satisfaction with pain control) and opioid consumption data were not available to directly assess prescribing appropriateness, although post-discharge healthcare utilization for pain-related concerns was evaluated.

As the intent of this study was to describe, in detail, discharge opioid exposure and dose intensity, with sampling based on hospital participation in NSQIP-Pediatric, differences in discharge opioid prescribing by provider were not assessed. The role of the prescribing provider on the healthcare team (eg, resident surgeon, advance practice providers, or attending surgeons), and the scope of their practice (adult and pediatric vs pediatric-only), has been shown to impact the intensity of opioid prescribing; however, these data were not available for this study.^32,36,37^ The participating sites included both free-standing children’s hospitals and pediatric programs embedded within larger adult healthcare systems, which may contribute to variation in prescribing practices. Analyses to explore the relevance of hospital type in explaining opioid prescribing variation may require a more qualitative study approach or a larger number of sites.

Finally, in this study, MMEs were used to compare opioid dose intensity and to identify higher-risk prescriptions.^34^ However, MMEs rely on adult-derived equianalgesic conversion factors and may not fully capture developmental differences in opioid pharmacology across pediatric populations. Age was included a priori as an independent variable in multivariable models based on clinical relevance to account for developmental variation. Nonetheless, further study of specific higher-risk procedures should also consider weight- and age-adjusted outcome measures to optimally improve comparability and address confounding.

## CONCLUSION

This multicenter, multispecialty, prospective, cohort study leveraged a national quality improvement platform and additional EHR-abstracted variables, revealing that approximately one-third of children in NSQIP-Pediatric are still prescribed an opioid at discharge, with significant variation across hospitals and specialties. The study supports the feasibility and value of NSQIP-Pediatric’s expansion to include specific pain-related EHR variables, facilitating future procedure-level analysis. Both discharge opioid exposure and dose intensity are critical metrics for optimal opioid stewardship.

## ACKNOWLEDGMENTS

Mallory N. Perez, MD, MS had full access to all data in the study and takes responsibility for the integrity of the data and the accuracy of the data analysis. Perez also conducted data cleaning, performed the statistical analyses, contributed to the interpretation of findings, and drafted the manuscript. Lynn Huang, the study biostatistician, also had full access to all data, contributed to data cleaning, performed statistical analyses, assisted in data interpretation, and revised the manuscript for critical content. Willemijn Schafer, PhD collaborated on conceptualization of the study, reviewed the analyses, and provided critical revisions of the manuscript. Alison J. Lehane, MD, MS and Charesa J. Smith, MD, MS contributed to the interpretation of results and reviewed and edited the manuscript. Sarah Kennedy, RN, MSN contributed to the study concept and design, providing critical input as a NSQIP-Pediatric SRC, reviewing the data and providing revisions of the manuscript. Jane L. Holl, MD, MPH contributed substantially to grant development, study design, data interpretation, and critically reviewed the manuscript. Charles J. Aprahamian, MD; Srikumar B. Pillai, MD; and Bethany J. Slater, MD, MBA, clinical principal investigators at three of the study’s participating sites, contributed to the study concept and design, oversaw data collection at their respective sites, participated in the interpretation of the data, and reviewed the manuscript. Mehul V. Raval, MD, MS oversaw the study in its entirety and assumes responsibility for the conception and design of the work; the acquisition, analysis, and interpretation of data; and critical revision of the manuscript for important intellectual content. All authors approved the final version of the manuscript and agree to be accountable for all aspects of the work.

We thank Susan Sullivan, MacKenton Johnson, and Victoria Bigdelle of University of Chicago – Comer University, Victoria Levin Sniegowski of Rush University, and Olivia Perham of OSF HealthCare Children’s Hospital of Illinois for their roles and clinical coordinators on this project and their expertise in NSQIP-Pediatric sampling and data collection.

## Supplementary Material
