# Supplementary material for: Safety Assessment of Perioperative Pain Medications for Children: Variation in Opioid Prescribing at Discharge
Source: Ann Surg Open. 2026 May 13;7(2):e664. doi: 10.1097/AS9.0000000000000664 (PMC13290244; doi:10.1097/AS9.0000000000000664)
Supplement: Supplementary file 1 [file as9-7-e664-s001.pdf]

Supplemental Table 1. List of ACS NSQIP-Pediatric & Pain-specific REDCap variables

**ACS NSQIP-PEDIATRIC**		**REDCap Variables**	
NSQIP-Pediatric ID		REDCAP ID	
Demographic data	Age	Opioid education provided	Y/N
	Gender/ethnicity	Local analgesic use	Type, concentration, volume
	Race	Regional blocks used	Y/N
	ASA class	Type of regional block	Epidural
			Nerve/TAP block
			Caudal
Type of procedure	Primary procedure (Current Procedural Terminology - CPT)	Medication administered in regional block	Type, concentration, volume
	Surgical specialty	Type of non-opioid used preoperatively	Acetaminophen, ketorolac, gabapentin
Operative approach	Laparoscopic/MIS only	Type(s) of opioid(s) prescribed	Hydrocodone, oxycodone, morphine, hydromorphone, codeine, tramadol
	Laparoscopic/MIS and Open		
	Open only or N/A	Details of opioid prescription	Concentration, volume, dose number
Surgical length of stay	Time from surgery date to date of discharge	Existing preoperative prescription for benzodiazepine	Y/N
30-day complications	Readmission	New benzodiazepine prescribed at discharge	Y/N
		Type of benzodiazepine	Name
	Return to operating room	Post-discharge pain-related issues	Opioid refill written within 30 days of discharge
	Other complications		Follow-up presentation for inadequately controlled pain (ED, urgent care, clinic, phone/email)

Supplemental Table 2. Percent Patients of Specialty Prescribed Opioids at Discharge by Site

Specialty	Overall	Hospital 1	Hospital 2	Hospital 3	Hospital 4	RMD	p-value
General Surgery (N=746)	111 (15%)	7 (5%)	8 (17%)	13 (10%)	83 (20%)	300 %	**<.001**
Orthopedics (N=312)	266 (85%)	51 (65%)	18 (100%)	31 (72%)	173 (95%)	54%	**<.001**
ENT (N=211)	44 (21%)	4 (19%)	14 (50%)	7 (23%)	19 (14%)	257 %	**0.001**
Neurosurgery (N=162)	29 (18%)	8 (40%)	0 (0%)	6 (25%)	15 (13%)	208 %	0.02
Urology (N=128)	36 (28%)	0 (0%)	0 (0%)	2 (10%)	34 (36%)	260 %	**0.004**
Plastics (N=84)	60 (71%)	1 (50%)	8 (67%)	10 (53%)	41 (80%)	60%	0.09
Gynecology (N=27)	20 (74%)	4 (40%)	3 (100%)	13 (93%)	0 (---)	150 %	0.008
Total (N=1670)	566 (34%)	72 (25%)	51 (45%)	82 (29%)	361 (37%)	80%	**<.001**

RMD: relative magnitude of difference

Supplemental Table 3. Percent Patients of Specialty Prescribed Opioids at Discharge by Age

Specialty	Overall	Children	Adolescents	RMD	p-value
General Surgery (N=746)	111 (15%)	14 (5%)	97 (22%)	340%	**<.001**
Orthopedics (N=312)	266 (85%)	90 (73%)	176 (94%)	29%	**<.001**
ENT (N=211)	44 (21%)	7 (7%)	37 (35%)	400%	**<.001**
Neurosurgery (N=162)	29 (18%)	9 (10%)	20 (26 %)	160%	0.009
Urology (N=128)	36 (28%)	21 (32%)	15 (24%)	33%	0.34
Plastics (N=84)	60 (71%)	25 (69%)	35 (73%)	6%	0.73
Gynecology (N=27)	20 (74%)	3 (75%)	17 (74%)	1%	1.0
Total (N=1670)	566 (34%)	169 (23%)	397 (42%)	82%	**<.001**

RMD: relative magnitude of difference
